# Dissecting the Causal Association Between Bulimia Nervosa and Structural Brain Abnormalities: A Two‐Sample Bidirectional Mendelian Randomization Study

**DOI:** 10.1002/brb3.70859

**Published:** 2025-09-10

**Authors:** Weihua Li, Xinghao Wang, Yiling Wang, Jiani Wang, Xinyu Huang, Marcin Grzegorzek, Qian Chen, Zhenchang Wang, Peng Zhang, Lirong Tang

**Affiliations:** ^1^ Department of Radiology, Beijing Friendship Hospital Capital Medical University Beijing China; ^2^ Department of Radiology Shengjing Hospital of China Medical University Shenyang China; ^3^ Department of Nuclear Medicine Shengjing Hospital of China Medical University Shenyang China; ^4^ Institute for Medical Informatics University of Luebeck Luebeck Germany; ^5^ German Research Center for Artificial Intelligence DFKI Lubeck Germany; ^6^ The National Clinical Research Center for Mental Disorders & Beijing Key Laboratory of Mental Disorders, Beijing Anding Hospital Capital Medical University Beijing China

**Keywords:** bulimia nervosa | causal relationships | Mendelian randomization (MR) | structural magnetic resonance imaging (MRI) phenotypes

## Abstract

**Background:**

Diverse correlations between structural brain abnormalities and the clinical feature of bulimia nervosa (BN) have been identified in previous observational studies.

**Objective:**

To explore the bidirectional causality between BN and brain structural magnetic resonance imaging (MRI) phenotypes.

**Methods:**

Genome‐wide association studies (GWAS) of 2441 participants identified genetic variants associated with disordered eating and predicted BN, whereas UK Biobank 3D‐T1 MRI data were used to analyze brain structural phenotypes. A bidirectional two‐sample Mendelian randomization (MR) approach was used to investigate the causal relationships between BN and brain structural traits.

**Results:**

The forward MR analysis showed that BN exerted a significant causal effect on decreased volume of left nucleus accumbens area (NAc), decreased surface area (SA) of left inferior temporal gyrus, and increased cortical thickness (CT) of left planum temporale and right inferior temporal gyrus. In the reverse MR analysis, we found that right putamen volume, left hippocampus volume, and right planum temporale gyrus CT were positively associated with BN risk. Besides, SA of right inferior temporal gyrus and left lateral orbital gyrus and CT of left superior occipital gyrus were inversely correlated with BN risk.

**Conclusion:**

Our findings confirmed the potential causal effects of BN on brain structure changes involving multiple functional regions and identified that genetically determined variation in specific brain structural regions could be causal for BN to some extent.

## Graphical abstract

This graphical abstract depicts the methodology of our two‐sample bidirectional Mendelian randomization analysis exploring causal associations between bulimia nervosa and brain structure.

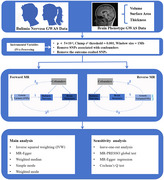

## 1 Introduction

Bulimia nervosa (BN) is a complex and severe psychiatric disorder characterized by recurrent episodes of binge eating followed by inappropriate compensatory behaviors, often resulting in substantial physical and psychological harm (Association 2013; Jahrami et al. 2024). Existing survey data suggest that the prevalence of eating disorders (ED) in China has been progressively increased in recent years (Yao et al. 2021). With estimates that 20%–30% of patients with anorexia nervosa or BN fail to respond to existing treatments and are likely to develop long‐term treatment‐refractory illnesses, and that only 30%–40% of BN patients achieve long‐term remission (Bryson et al. 2024; Mairs and Nicholls 2016; Treasure et al. 2020). Extensive research has been conducted on BN in the fields of genetics, microbiology, and neurocognition. These challenges highlight the need for a more precise understanding of the underlying neurobiological mechanisms to inform the development of targeted interventions.

In recent years, numerous observational studies have been conducted to investigate the relationships between brain imaging‐derived phenotypes and BN. Structural magnetic resonance imaging (MRI), a vital technique for investigating neuroimaging characteristics, enables the extraction of key metrics such as regional volume, surface area (SA), and cortical thickness (CT), offering valuable insights into their associations with BN. Different brain regions play distinct roles in regulating behavior and performance. Observational studies have identified various associations between neuroimaging abnormalities in specific brain regions and core features of BN, such as impaired impulse control and maladaptive eating patterns (Berner et al. 2023; W. Li et al. 2023, 2024; J. Wang et al. 2023; Y. Wang et al. 2023). It has been reported, for instance, that patients with BN exhibit increased volumes in the medial orbitofrontal cortex (mOFC), ventral striatum, and insula, which may be linked to dysfunctions in food reward processing and/or self‐regulation (W. H. Li et al. 2022; Schafer et al. 2010). However, other studies have reported a reduction in grey matter volume (GMV) in the superior frontal gyrus (SFG), superior temporal gyrus (STG), median cingulate and paracingulate gyri (DCG), as well as in the caudate and putamen (Coutinho et al. 2015; Frank et al. 2013; X. Li et al. 2020). Furthermore, studies from surface‐based morphometry (SBM) approaches demonstrate reductions in SA in the frontal and temporoparietal regions, as well as decreased CT in the pars triangularis, superior parietal gyrus, and dorsal posterior cingulate cortex in patients with BN (Berner et al. 2018; Marsh et al. 2015). Although our SBM analyses revealed greater sulcal depth in the STG and mOFC, they did not show any alterations in CT, SA, volume, or mean curvature (W. Li et al. 2023). These inconsistencies across observational studies underline the inherent limitations of such research designs in establishing clear causal relationships between brain structural alterations and BN. On the other hand, observational studies are often subject to confounding factors, reverse causality, and biases, which make it challenging to determine whether the observed neuroimaging changes are a cause or consequence of BN. Given these complexities, a robust method that allows for the assessment of causal links is essential.

Mendelian randomization (MR) is a statistical strategy that uses Mendelian inheritance rules to do directional analysis (Davies et al. 2018). MR is a useful data analysis approach for assessing etiological inference in epidemiological research. It employs genetic variation as an instrumental variable (IV) to determine the causal link between the exposure factor of interest and the result of interest. The data used for this analysis comes from genome‐wide association studies (GWAS) with the same genetic background. This strategy leverages the effect of randomly assigned genes in nature on phenotypes to deduce the influence of phenotypes on disease or symptoms. As MR analysis is based on genetic variation, which is unaffected by common confounding variables, the process is referred to as randomization, which is thought to prevent confounding factors and yield actual causal associations (Sekula et al. 2016). At the same time, causality is appropriate for investigating the relationship between brain imaging traits and symptoms. Observational or retrospective studies frequently struggle to explain the direction of the impact, whereas MR can have a clear causal direction, and several studies have attempted (Seyedsalehi et al. 2023; Wang et al. 2024). Therefore, employing the benefits of MR to investigate the impact of BN on brain structure is extremely congruent with the disease's actual alterations and treatment approaches.

In summary, the goal of this study is to explore whether there is evidence supporting the hypothesis that brain structural alterations lie on the causal pathway of BN, utilizing T1 imaging in conjunction with MR protocols. We used imaging markers of grey matter from structural MRI as proxies for the anatomical structure and morphology characteristics of the brain. Our primary objectives were (i) to assess whether BN has a causal effect on brain grey matter structure and (ii) to assess whether brain grey matter structural phenotypes causally affect risk of BN.

## 2 Materials and Methods

### 2.1 Study Design

**FIGURE 1:**
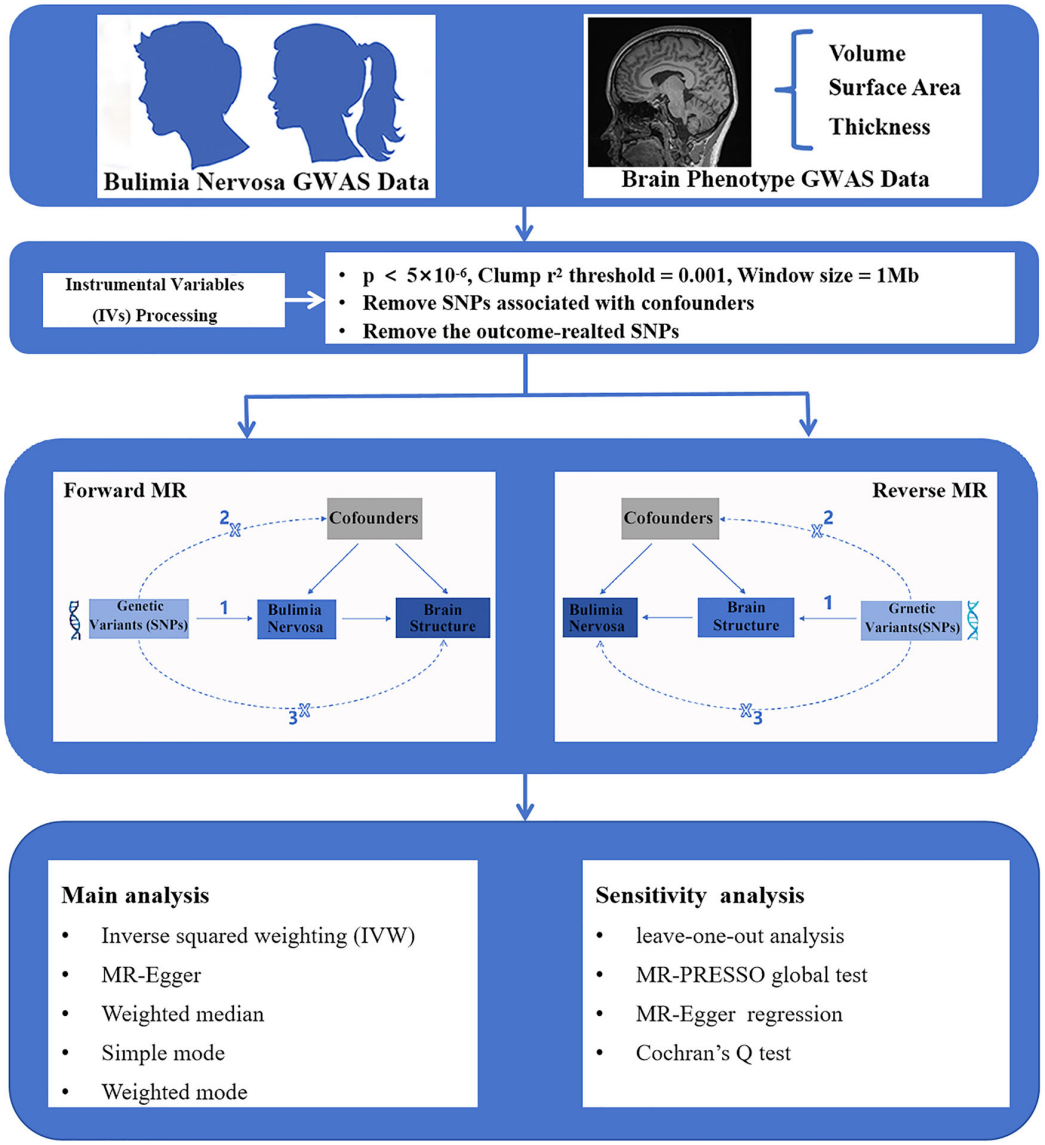
Workflow of bidirectional Mendelian randomization analysis between bulimia nervosa and brain structural phenotypes. GWAS, genome‐wide association studies; MR, Mendelian randomization; SNPs, single‐nucleotide polymorphisms.

This study was conducted in accordance with the STROBE‐MR statement to ensure rigorous adherence to MR analysis protocol (Skrivankova et al. 2021). An overview of the study design and MR analysis workflow is presented in Figure 1. Briefly, our study commenced with an assessment of genetic correlations between BN and specific brain structural phenotypes. Single‐nucleotide polymorphisms (SNPs) showing independence and significant associations with exposure were retained as IVs, whereas those associated with confounding factors were excluded. Following this, a bidirectional two‐sample MR analysis was conducted to infer the causality between BN and brain structural phenotypes. Sensitivity analyses were subsequently conducted to assess the robustness of the observed causal effects.

### 2.2 GWAS Data

#### 2.2.1 GWAS Data Sources for BN

To investigate the genetic association of dietary disorders, a study reported the results of genome‐wide association analysis of 2564 pairs of twins (Wade et al. 2013). Among these participants, BN is a major focus, with genotype data from 2442 individuals and 11,846,513 SNPs. The genotype is derived from the current QIMR genetic epidemiology laboratory GWAS data, which incorporates eight batches of genotyping data collected using standard Illumina chips and involves almost 19,000 people (made up of twins, core families, or monomers). In each batch, use the genotyping module in BeadStudio to call genotypes and then perform data cleaning. The study analyzed 7,262,007 SNPs (only *R* ^2^ quality control testing was performed), filtered out SNPs with minor allele frequency (MAF) <2%, and got the final GWAS findings represented by BN depending on various symptoms. For more details about BN, please refer to Supporting Information1.

#### 2.2.2 GWAS Data Sources for BN

The representative parameters of brain structure include the volume, SA, and CT of the entire brain or region. These parameters come from 3D‐T1 images (T2 participates in registration), and all these resources come from UK Biobank (Elliott et al. 2018; Smith et al. 2021). External ethical committees have approved all the UK Biobank's data‐gathering procedures. 3D‐T1's image resolution was 1 × 1 × 1 mm^3^. The image's field of view was 208 × 256 × 256 matrix (scan parameters: 3D MPRAGE, pre‐scan normalization). After eliminating any non‐brain tissue, the image is placed into the connection using the MNI152 “nonlinear generation 6” standard space T1 template. The brain anatomy was segmented using the Harvard Oxford cortical and subcortical atlas, as well as the Dietrichsen cerebellar atlas. Subcortical structures (in the form and volume) were modeled using the tool (FMRIB's integrated registration and segmentation program) (Patenaude et al. 2011). The study investigated the association between brain imaging parameters and genes and performed corrections (at a threshold of −log10 (*P*) > 11), which additionally corrected for all 3144 GWAS carried out, resulting in 165 indicators related to brain structure (specific indicators can be found in Supporting Information 2).

### 2.3 IVs Selection

In accordance with the core assumptions of MR analysis, we applied a systematic filtering procedure to select eligible IVs: (i) SNPs significantly associated with the exposure were identified using a threshold of *p* < 5 × 10^−6^; (ii) independent SNPs were obtained through linkage disequilibrium (LD) clumping with a window size of 1 Mb and an *r* ^2^ threshold of 0.001; (iii) SNPs associated with potential confounding factors.

### 2.4 MR Analysis and Quality Control

All MR statistical analysis and data visualization were performed with R software (version 4.2.2) and the “Two‐Sample‐MR packages” (https://github.com/MRCIEU/Two‐Sample‐MR) (Yavorska and Burgess 2017).

This study is a two‐sample MR study based on brain imaging parameters and BN. To determine whether BN can affect brain structure, we first selected closely related SNPs from the GWAS results of BN. In this process, after matching and coordinating the selected representative SNPs with the outcome variables (brain structure‐related neuroimaging indicators), a variety of MR methods (mainly inverse‐variance weighting [IVW]) were analyzed (Burgess, Butterworth, and Thompson 2013). To account for mild IV bias, the usual genome‐wide significance (threshold 5 × 10^−6^) was used as a sensitivity study. Five high‐efficiency approaches were used to evaluate causal effects: the IVW technique (which served as the fundamental MR method), simple mode, MR‐Egger regression (sensitivity analysis), weighted median method, and MR pleiotropy residual sum and outlier (MR‐PRESSO) method. In addition, the results of MR were tested for heterogeneity among the SNPs included in each analysis using Cochran's *Q*‐test. If there is statistical significance (*p* value less than 0.05), heterogeneity exists, and vice versa. The detection of horizontal pleiotropy relies on the intercept of MR Egger to evaluate the instrument strength hypothesis that is independent of direct effects. We consider the final *p* value to be meaningful if it is less than 0.05 on both sides. For reverse MR, we use brain imaging parameters as the exposure and BN as the outcome to explore the impact of brain changes on the occurrence of BN. Of course, the analysis process of reverse MR is consistent with the forward analysis process mentioned above.

## 3 Results

### 3.1 Overview of the Study

**FIGURE 2:**
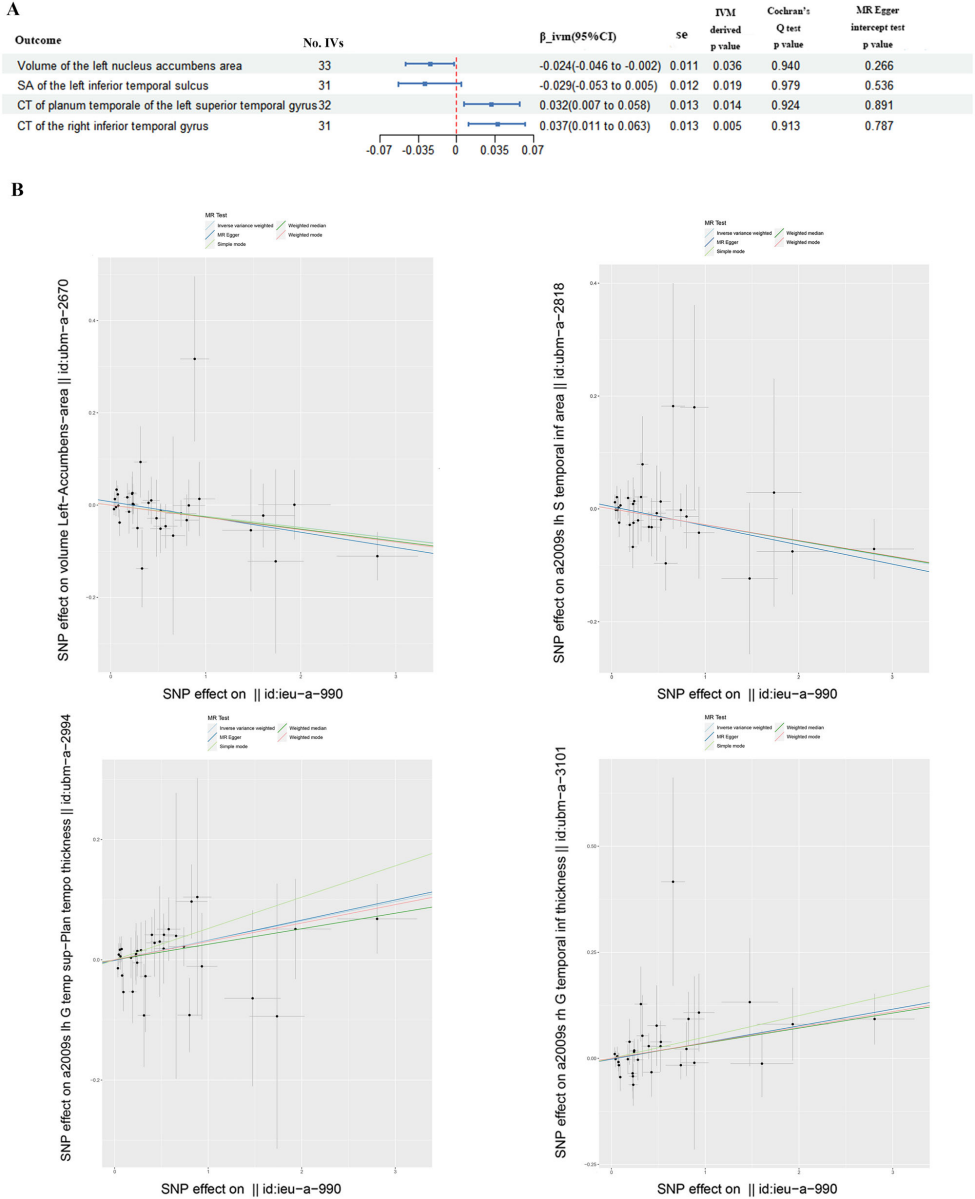
Significant findings from the forward Mendelian randomization (MR) analysis of bulimia nervosa on brain structural traits. And *p* < 0.05 was regarded as suggestive potential causality. (A) Four brain structural traits related to the risk of bulimia nervosa in the forward MR analysis. (B) Five analysis methods in the forward MR analysis of the above brain structural traits. CI, confidence interval; CT, cortical thickness; IVW, inverse‐variance weighted; No. IVs, number of instrumental variables; SA, surface area; se, standard error; SNP, single‐nucleotide polymorphisms.

**FIGURE 3:**
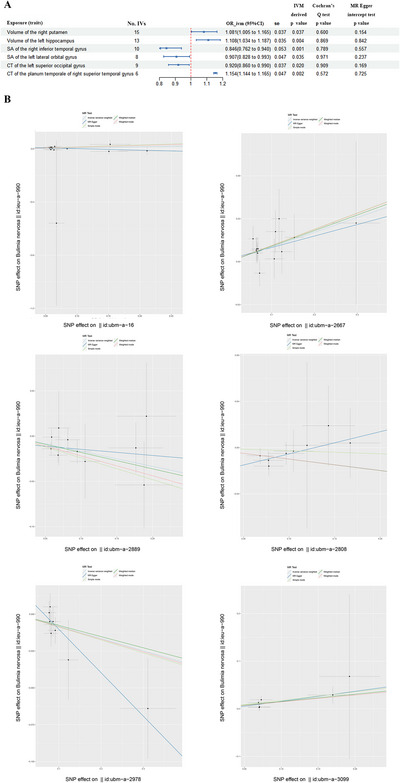
Significant findings from the reverse Mendelian randomization (MR) analysis of bulimia nervosa on brain structural traits. And *p* < 0.05 was regarded as suggestive potential causality. (A) Six brain structural traits associated with the risk of bulimia nervosa in reverse MR analysis. (B) Five analysis methods in the reverse MR analysis of the above six structural traits. CI, confidence interval; CT, cortical thickness; IVW, inverse‐variance weighted; No. IVs, number of instrumental variables; OR, odds ratio; SA, surface area; se, standard error; SNP, single‐nucleotide polymorphisms.

On the basis of the predefined criteria for selecting IVs, 31–33 SNPs were included in the forward MR analysis, whereas 6–15 SNPs were retained in the reverse MR analysis. Detailed summaries of the SNPs used in the forward and reverse MR analyses are provided in Supporting Information 3 and 4, respectively. Following the bidirectional MR analyses, we identified four and six brain structural traits as potential outcomes and risk factors for BN, respectively (IVW‐derived *p* value <0.05; Figures 2A and 3A). As shown in Figures 2A and 3A, all significant associations passed quality control assessments, showing no evidence of heterogeneity or horizontal pleiotropy. Additional analyses using the MR‐Egger, simple mode, weighted median, and weighted mode methods yielded consistent causal estimates in the same direction (Figures 2B and 3B). More detailed results of the bidirectional MR analyses are presented in Supporting Information 5. Supporting Information 6 provides MR result plots for each exposure, including comparisons across different MR methods (Figure A series), funnel plots (Figure B series), single‐SNP MR forest plots (Figure C series), and leave‐one‐out sensitivity analyses (Figure D series).

### 3.2 Forward MR Analysis of BN on Brain Structural Traits

#### 3.2.1 Causal Evaluations of Genetically Predicted BN on Volume

The IVW‐based MR (IVW‐MR) analysis showed that the susceptibility to BN was potentially associated with the reduced volume in the left nucleus accumbens area (NAc) (*β*_ivm_ = −0.024 mm^3^ [95% confidence interval (CI): −0.046, −0.002], *p* = 0.036).

#### 3.2.2 Causal Evaluations of Genetically Predicted BN on Cortical SA

We found a significant association between genetically predicted BN and the decreased cortical SA in the left inferior temporal gyrus (IVW‐MR: *β*_ivm_ = −0.029 mm^2^ [95% CI: −0.053, −0.005], *p* = 0.019).

#### 3.2.3 Causal Evaluations of Genetically Predicted BN on CT

The genetically predicted BN was nominally associated with increased CT in the left planum temporale gyrus (*β*_ivm_ = 0.032 mm [95% CI: 0.007, 0.058], *p* = 0.014) and the right inferior temporal gyrus (*β*_ivm_ = 0.037 mm [95% CI: 0.011, 0.063], *p* = 0.005).

### 3.3 Reverse MR Analysis of Brain Structural Traits on BN

#### 3.3.1 Grey Matter Volume and BN Risk

In the reverse MR analysis, we found that the volume of the right putamen and the left hippocampus were positively associated with BN risk. The IVW analysis results indicated that the probability of BN was, respectively, increased by 8.1% (odds ratio (OR_ivw_) = 1.081 [95% CI: 1.005–1.165], *p* = 0.037), 10.8% (OR_ivw_ = 1.108 [95% CI: 1.034–1.187], *p* = 0.004) with one‐standard deviation (1‐SD) increase in the left hippocampus and the right putamen.

#### 3.3.2 Cortical SA and BN Risk

The two‐sample MR analysis also found that the SA of the right inferior temporal gyrus (OR_ivw_ = 0.846 [0.762–0.940], *p* = 0.001) and the left lateral orbital gyrus (OR_ivw_ = 0.907 [95% CI: 0.828–0.993], *p* = 0.035) was inversely associated with BN risk.

#### 3.3.3 CT and BN Risk

Besides, we also found that the CT of left superior occipital was inversely associated with BN risk (OR_ivw_ = 0.92 [95% CI: 0.86–0.99], *p* = 0.026) and the right planum temporale CT was positively associated with BN risk (OR_ivw_ = 1.154 [95% CI: 1.144–1.165], *p* = 0.002).

### 3.4 Sensitivity Analyses

We conducted a series of sensitivity analyses to corroborate the putative causal relationships between BN and brain structure changes obtained from bidirectional MR. First, leave‐one‐out analyses revealed that no single SNP influenced the causal estimates. The detailed results of the leave‐one‐out sensitivity analysis are presented in Supporting Information 6 (Figure D series). Second, the MR‐Egger intercepts of all associations were found in close proximity to zero, suggesting the absence of significant pleiotropy. Third, the directions of the association from other MR methods were the same as those of the IVW method, which supports the reliability of our inferred causal effects. Overall, the sensitivity analyses confirmed the reliability of our putative causal effects in both the forward and reverse MR results.

## 4 Discussion

We conducted bidirectional univariable MR analyses using large genome‐wide association datasets to examine putative causal relationships between brain macrostructure, including volume, cortical SA, and thickness, as measured by structural MRI, and risk of BN. We found strong evidence in support of interactions between BN and several measures of specific brain regions involved in the reward system, cognitive functions, and visuoperceptual functions, as well as emotional and memory functions. These findings provide insights into the neurobiological mechanisms of BN and offer a theoretical basis for neuroregulation strategies in future clinical treatments.

According to our results, there was a causal effect of genetically predicted BN on the volume of left NAc. The NAc, as a major component of the ventral striatum, serves as a critical node within the brain's reward circuitry and has long been regarded as a key structural foundation for the regulation of motivated behaviors and reward, particularly playing a central role in the regulation of food intake (Berland et al. 2021; Vaseghi et al. 2022). Additionally, the NAc is essential for action selection, combining cognitive and emotional inputs from the frontal and temporal lobes to improve the efficiency and strength of behaviors motivated by rewards or aversive stimuli (Floresco 2015). Furthermore, recent research on guilt subtypes demonstrates that normative guilt correlates with bingeing/purging behaviors in individuals with ED, whereas altruistic guilt predicts heightened interpersonal distrust (Raffone et al. 2025). Growing evidence supports the involvement of the NAc in regulating unhealthy eating habits and suggests its potential role in the neurobiological basis of ED such as anorexia nervosa, obesity, and BN (Domingo‐Rodriguez et al. 2020; Leppanen et al. 2020; Rapuano et al. 2020; Volkow et al. 2013). In BN, previous studies have observed that, compared to healthy subjects, the total volume of the NAc remains preserved, but there are inward deformations on the surface of the NAc (Berner et al. 2019; Coutinho et al. 2015; Y. Wang et al. 2023). Complementarily, researchers have identified abnormal neural activation and functional network reorganization in different subregions of the NAc in BN, with the reorganization primarily occurring in the frontal cortex and being associated with emotional eating behavior (Y. Wang et al. 2023). These findings are partially inconsistent with our results, possibly due to limitations in the study sample, differences in diagnostic criteria, disease duration, severity of clinical symptoms, and comorbidities. Our MR analysis provides additional evidence by indicating a causal association between BN and reduced volume in the left NAc. Structural brain alterations of the NAc observed in our MR study may map onto specific motivated and reward‐related processes in BN. The NAc may serve as a potential neurobiological marker for BN, potentially emerging after the onset of symptoms.

In the current study, we identified evidence for bidirectional causal relationships between BN and morphological changes in the temporal lobe. Specifically, there were causal associations of genetically predicted BN on the decreased SA of left inferior temporal gyrus and the increased CT of left planum temporale and right inferior temporal gyrus, whereas an increase in the CT of the right planum temporale and a decrease in the SA of right inferior temporal gyrus were associated with higher BN risk. The temporal lobe plays a significant role in visual perception, particularly in the higher order processing of visual information. Some scholars believe that the reduced STG volume in BN may be associated with distorted body image perception, potentially contributing to restrictive eating or binge eating behaviors (Donnelly et al. 2018; Kohmura et al. 2017; X. Li et al. 2020). Our previous study also found an increased sulcal depth in the right STG of BN relative to healthy controls (W. Li et al. 2023). Other studies have also detected reductions of SA and CT in local temporal regions (Marsh et al. 2015; Westwater et al. 2018). Overall, these findings provide valuable insights into the complex interactions between BN and the morphological changes of temporal lobe involved in visual and cognitive processing. Unfortunately, we note that when bidirectional MR analysis indicates causal relationships in both directions, it is challenging to determine whether these truly represent reciprocal causality or may reflect shared etiological factors. Such shared etiological factors may include neurodevelopmental vulnerabilities, early life environmental exposures, or pleiotropic genetic effects governing both brain structure and BN susceptibility.

In our reverse MR analysis, we identified significant associations between specific brain structural variations and BN risk. Notably, we identified three brain structural traits that potentially contribute to the risk of BN, including the right putamen GMV, the left hippocampus GMV, and the right planum temporal CT. The increased GMV in the left hippocampus, a region crucial for memory formation and emotional regulation, may reflect alterations in neuroplasticity or neurodevelopmental processes that predispose individuals to BN. The hippocampus is known to be involved in the processing of emotional and contextual information, which could influence the development of disordered eating behaviors (Davidson et al. 2009). The previous research has found that BN patients showed hypoactivation during reorienting and executive attention in *para*‐hippocampus compared with controls (Seitz et al. 2016). The increased GMV observed in the right putamen, a key component of the basal ganglia involved in reward processing and motor control, suggests a possible dysregulation in the brain's reward system. Similarly, Amianto et al. (2013) found BN patients showed a greater GMV in the left putamen, compared with healthy controls, which may be associated with triggering unhealthy eating behaviors. These findings align with the notion that individuals with BN might experience altered reward sensitivity, which could manifest as heightened reward responses to food‐related stimuli or compensatory behaviors. In addition, three brain structural traits potentially linked to a lower risk of BN were identified, including the left superior occipital CT, the SA of the right inferior temporal gyrus, and the left lateral orbital gyrus. The orbital cortex, an area implicated in decision‐making and social cognition, plays an important role in food intake control and satiety regulation (Monteleone et al. 2018; Plassmann et al. 2010). The orbital cortex is also involved in higher order cognitive processes, including the ability to adaptively shift strategies and make flexible decisions based on changing environmental cues. The decreased SA in the left lateral orbital cortex might reflect diminished cognitive flexibility or impaired executive functions, which could affect an individual's ability to plan and execute complex behaviors related to eating, such as portion control or resisting impulsive eating. Furthermore, the occipital lobe is related to the perception and processing of visual information, as well as the organization of complex visual perception processes (Donnelly et al. 2022). And visual processing is an important input interface to reward circuitry. Enhanced CT in the left superior occipital gyrus may strengthen top‐down regulation of food‐related visual cues or mitigate body image distortions—core vulnerabilities in BN—potentially conferring resilience against dysregulated eating behaviors. These findings suggest that alterations in brain regions involved in memory, reward processing, decision‐making, and visual perception may contribute to heightened susceptibility to BN, potentially through dysregulation in neuroplasticity, reward sensitivity, and cognitive flexibility.

However, this study also has some limitations, such as: (1) The MR method itself should be discussed first. It has been suggested that the strength of evidence provided by MR for epidemiological conditions is considered second only to meta‐analyses (Davey Smith et al. 2020). Nevertheless, the MR approach itself is susceptible to a wide range of influences, including weak variables, the level of GWAS data quality, and other related difficulties. Although we conducted some tests to prevent weak variables and pleiotropy from affecting the results, MR results are best explored in conjunction with observational studies to uncover true causal associations; (2) one drawback that should be noted is the relatively moderate amount of GWAS studies for BN. Although the genetic validity of this GWAS is adequate, the sample size is expected to be increased further due to a lack of the most recent relevant data; (3) some argue that BN is a variation of anorexia, and additional investigation of the two combined would be more compelling in determining genetic or causal links between them; (4) we should pay attention to the gender differences and accompanying psychological symptoms of BN. We excluded SNPs related to outcomes but did not exclude SNPs related to the aforementioned factors, so further multivariate MR analysis should be conducted to explore; (5) then, the genetic background of this study is in Europe, which may limit the generalizability of the findings to other ancestral groups. Future studies are encouraged to incorporate more ancestrally diverse cohorts and conduct cross‐ancestry MR analyses to improve the robustness and transferability of the results.

## 5 Conclusions

Our study revealed novel evidence of causal relationships between BN and structural changes in brain regions associated with the reward system, cognitive functions, visuoperceptual processes, and emotional and memory functions. These findings contribute important understanding of the neurobiological mechanisms involved in BN and lay the groundwork for potential neuroregulation approaches in future clinical interventions.

## Author Contributions

**Weihua Li**: writing – original draft. **Xinghao Wang**: writing – original draft. **Yiling Wang**: data curation, software, visualization. **Jiani Wang**: software, data curation, visualization. **Xinyu Huang**: methodology, software. **Marcin Grzegorzek**: methodology, software. **Qian Chen**: methodology, validation. **Zhenchang Wang**: project administration, supervision. **Peng Zhang**: supervision, writing – review and editing. **Lirong Tang**: supervision, writing – review and editing.

## Disclosure

Patients or the public were not involved in the design, or conduct, or reporting, or dissemination plans of our research.

## Ethics Statement

This study only used published and publicly available data. Ethical approvals referenced in the investigation, including informed consent from each participant, can be found in the original publications.

## Consent

The authors have nothing to report.

## Conflicts of Interest

The authors declare no conflicts of interest.

## Peer Review

The peer review history for this article is available at https://publons.com/publon/10.1002/brb3.70859.

## Supporting information




**Supplementary Material**: brb370859‐sup‐0001‐SuppMat1.docx

**Supplementary Material**: brb370859‐sup‐0002‐SuppMat2.xlsx

**Supplementary Material**: brb370859‐sup‐0003‐SuppMat3.xlsx

**Supplementary Material**: brb370859‐sup‐0004‐SuppMat4.xlsx

**Supplementary Material**: brb370859‐sup‐0005‐SuppMat5.xlsx

**Supplementary Material**: brb370859‐sup‐0006‐SuppMat6.pdf

## Data Availability

All data are publicly accessible. The GWAS datasets are sourced from IEU‐OpenGWAS (https://gwas.mrcieu.ac.uk/). For detailed information on the brain imaging resources and data processing methods, please consult the official UK Biobank Brain Imaging website: UK Biobank Brain Imaging (https://www.fmrib.ox.ac.uk/ukbiobank/index.html).
