# Supplementary material for: Dissecting the Causal Association Between Bulimia Nervosa and Structural Brain Abnormalities: A Two‐Sample Bidirectional Mendelian Randomization Study
Source: Brain Behav. 2025 Sep 10;15(9):e70859. doi: 10.1002/brb3.70859 (PMC12423433; doi:10.1002/brb3.70859)
Supplement: Supplementary file 1 — Supplementary Material: brb370859‐sup‐0001‐SuppMat1.docx [file BRB3-15-e70859-s001.docx]

**GWAS description of BN**


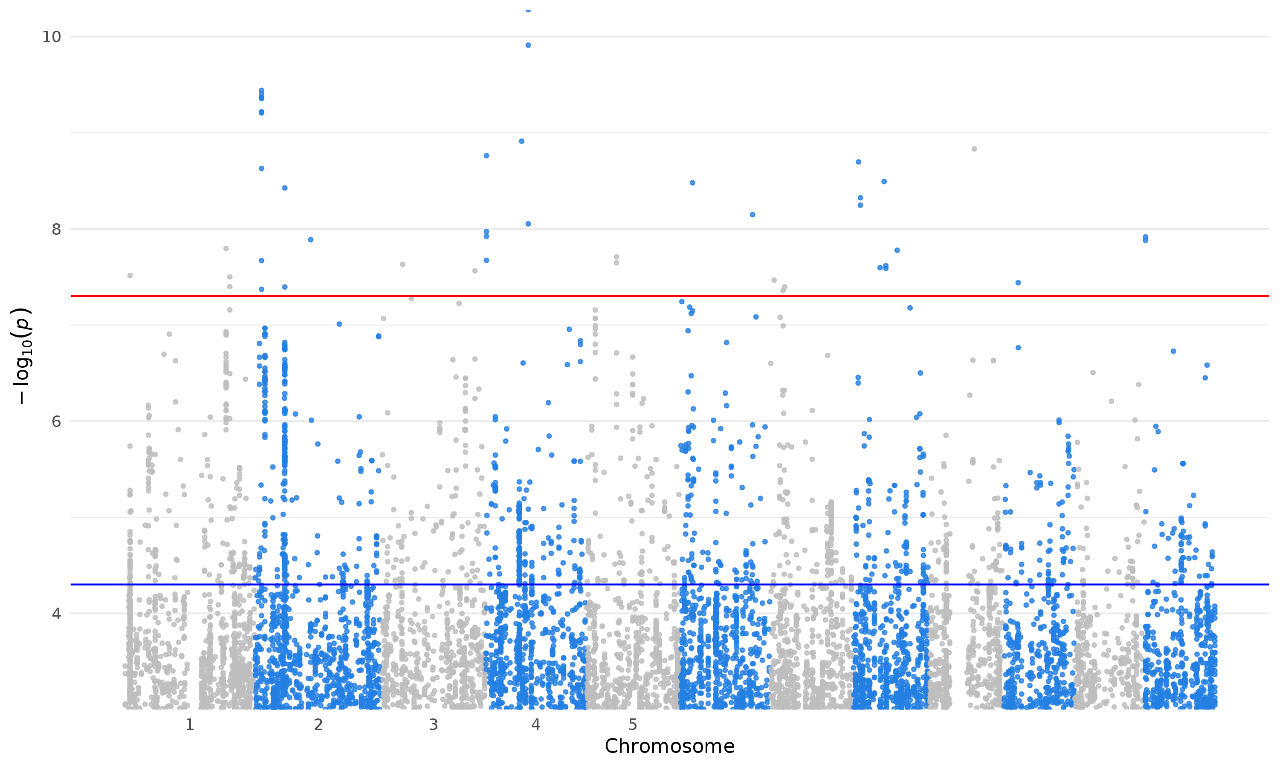


Manhattan plots: 1000 Genomes-based dosage scores (SNPs with R2>0.3& MAF>0.02) for the BN


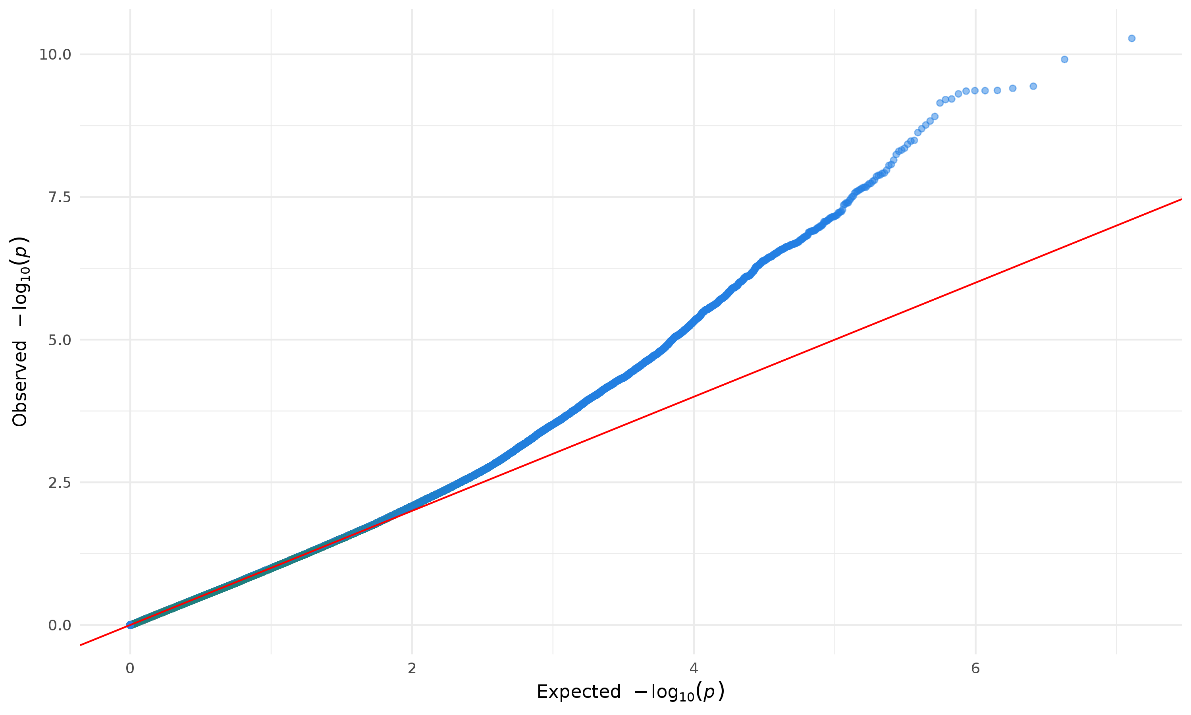


QQ Plot for the BN

**LD Score Regression (LDSC):**

Total Observed scale h2: 0.4385 (0.1898)

Lambda GC: 1.0432

Mean Chi^2: 1.0181

Intercept: 0.9973 (0.007)

Ratio < 0 (usually indicates GC correction).
