# Supplementary material for: Dissecting the Causal Association Between Bulimia Nervosa and Structural Brain Abnormalities: A Two‐Sample Bidirectional Mendelian Randomization Study
Source: Brain Behav. 2025 Sep 10;15(9):e70859. doi: 10.1002/brb3.70859 (PMC12423433; doi:10.1002/brb3.70859)

Catalogue

1. SNP effect on volume Left−Accumbens−area || id:ubm−a−2670.........1

2. SNP effect on a2009s lh S temporal inf area || id:ubm−a−2818............2

3. SNP effect on a2009s lh G temp sup−Plan tempo thickness || id: ubm −a−2994...3

4. SNP effect on a2009s rh G temporal inf thickness || id:ubm−a−3101...4

5. SNP effect on Bulimia nervosa || id:ubm-a-16 || id:ieu-a-990...............5

6. SNP effect on Bulimia nervosa || id:ubm-a-2667 || id:ieu-a-990...........6

7. SNP effect on Bulimia nervosa || id:ubm-a-2808 || id:ieu-a-990...........7

8. SNP effect on Bulimia nervosa || id:ubm-a-2889 || id:ieu-a-990...........8

9. SNP effect on Bulimia nervosa || id:ubm-a-2978 || id:ieu-a-990...........9

10. SNP effect on Bulimia nervosa || id:ubm-a-3099 || id:ieu-a-990.......10

Note: The MR analysis result plots for each exposure show the comparison of results using different MR methods (Figure A), funnel plot (Figure B), forest plot of single SNP MR (Figure C) and leave-one-out sensitivity analysis result (Figure D) for that exposure, respectively.

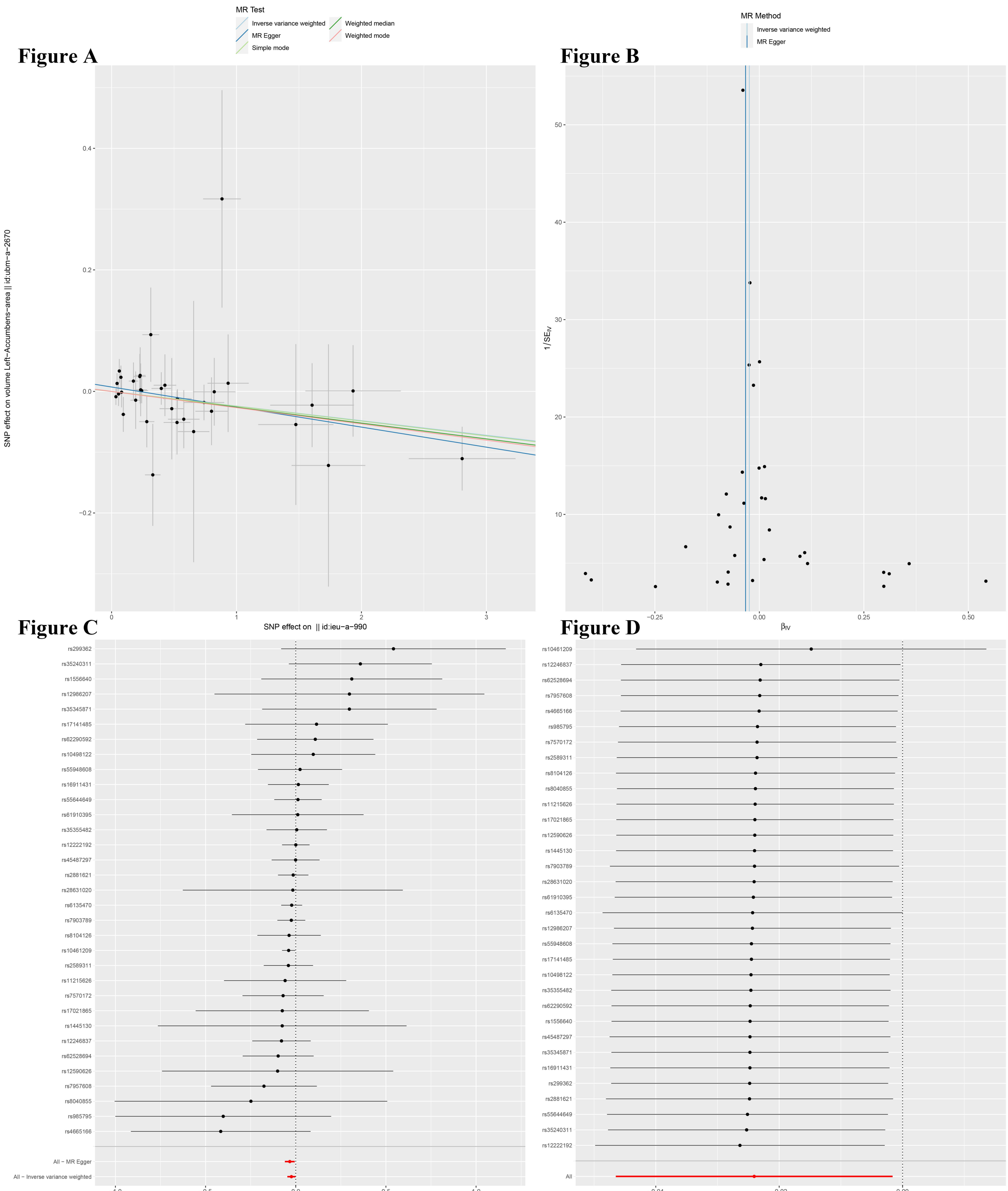

Figure A
MR Test
Inverse variance weighted
MR Egger
Simple mode
Weighted median
Weighted mode
SNP effect on volume Left−Accumbens−area || id:ubm−a−2670
SNP effect on || id:ieu−a−990
Figure B
MR Method
Inverse variance weighted
MR Egger
$1/SE_{IV}$
β_{IV}
Figure C
rs299362
rs35240311
rs1556640
rs12986207
rs35345871
rs17141485
rs62290592
rs10498122
rs55948608
rs16911431
rs55644649
rs61910395
rs35355482
rs12222192
rs45487297
rs2881621
rs28631020
rs6135470
rs7903789
rs8104126
rs10461209
rs2589311
rs11215626
rs7570172
rs17021865
rs1445130
rs12246837
rs62528694
rs12590626
rs7957608
rs8040855
rs985795
rs4665166
All − MR Egger
All − Inverse variance weighted
MR effect size for
' || id:ieu−a−990' on 'volume Left−Accumbens−area || id:ubm−a−2670'
Figure D
rs10461209
rs12246837
rs62528694
rs7957608
rs4665166
rs985795
rs7570172
rs2589311
rs8104126
rs8040855
rs11215626
rs17021865
rs12590626
rs1445130
rs7903789
rs28631020
rs61910395
rs6135470
rs12986207
rs55948608
rs17141485
rs10498122
rs35355482
rs62290592
rs1556640
rs45487297
rs35345871
rs16911431
rs299362
rs2881621
rs55644649
rs35240311
rs12222192
All
MR leave−one−out sensitivity analysis for
' || id:ieu−a−990' on 'volume Left−Accumbens−area || id:ubm−a−2670'

1

Figure A

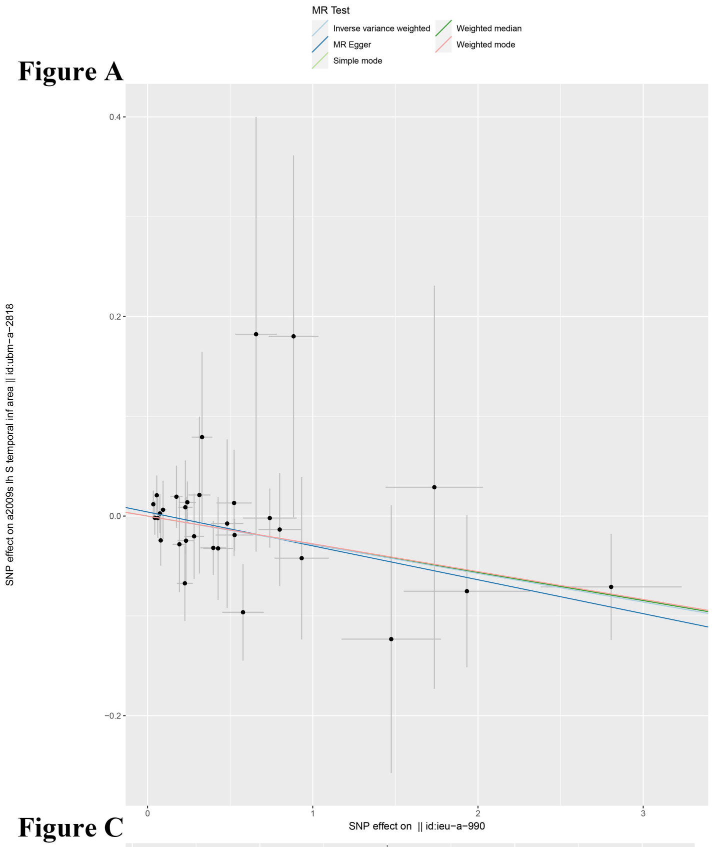

Figure B

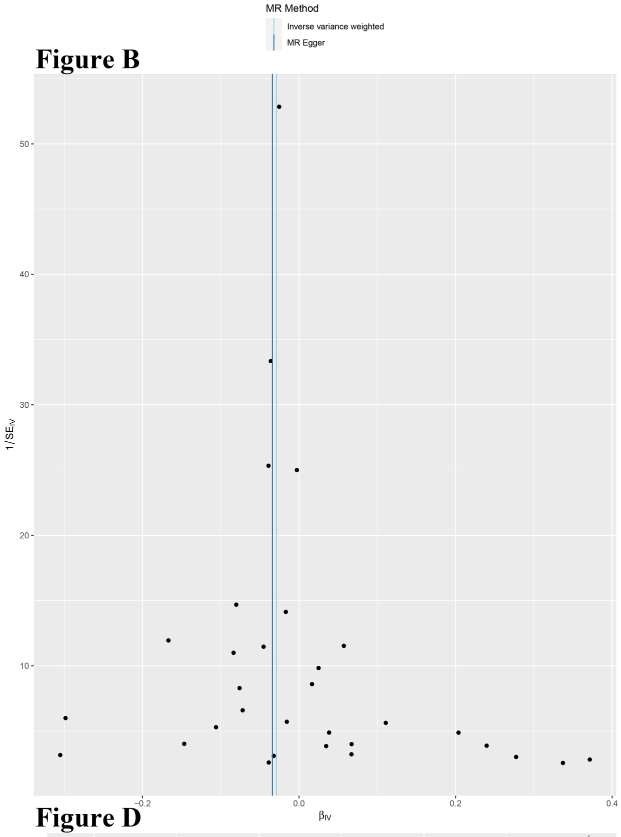

Figure C

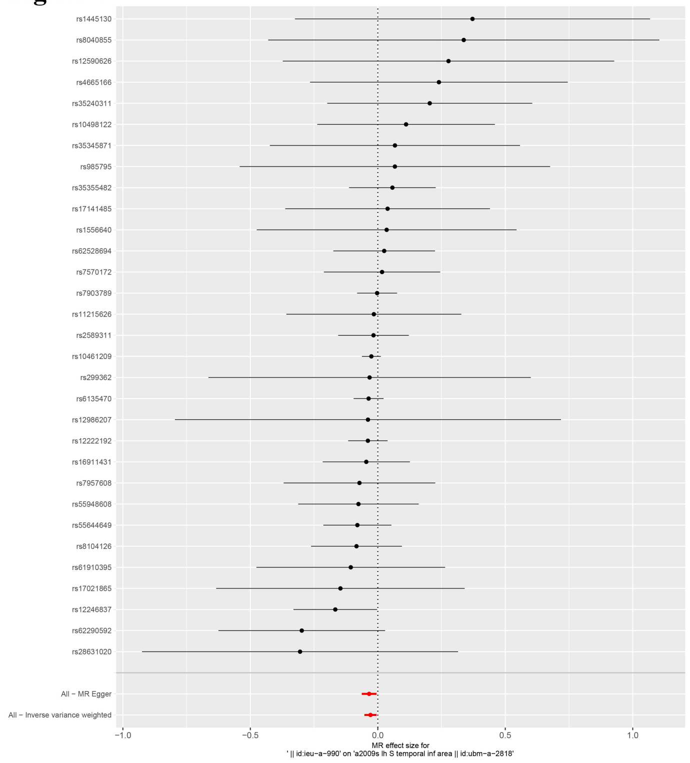

Figure D

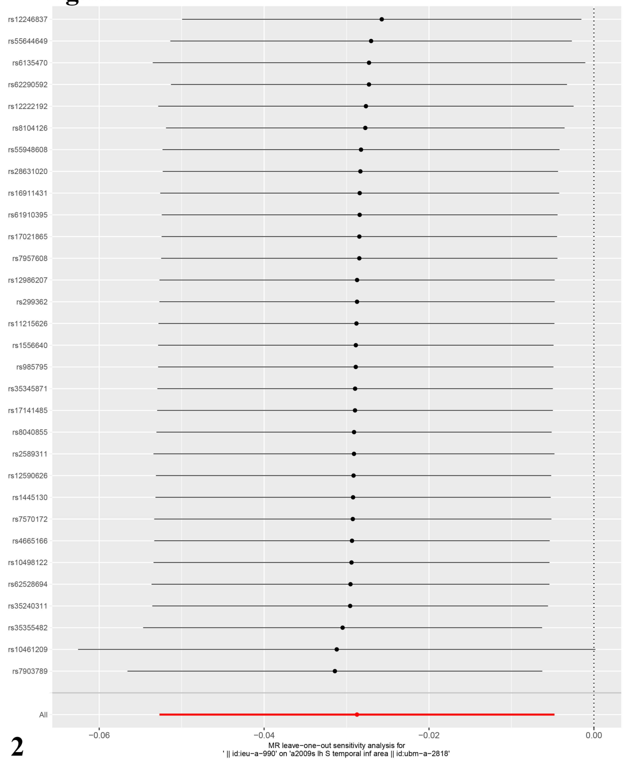

2

Figure A

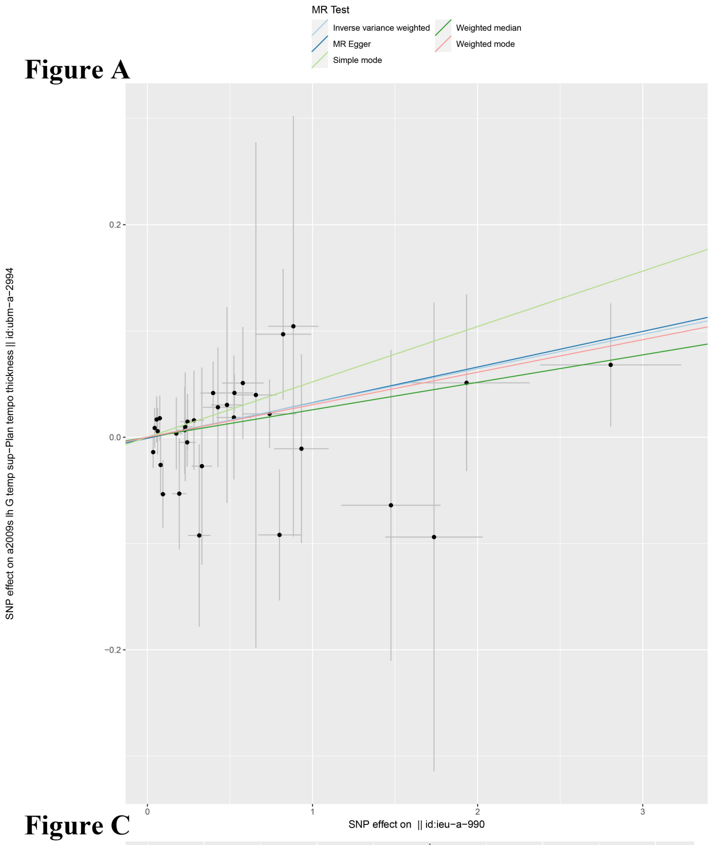

Figure B

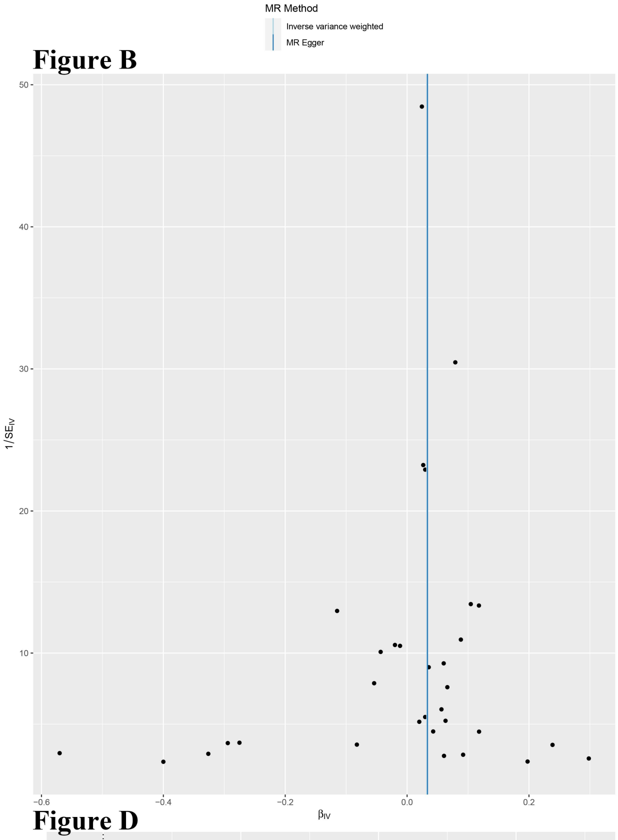

Figure C

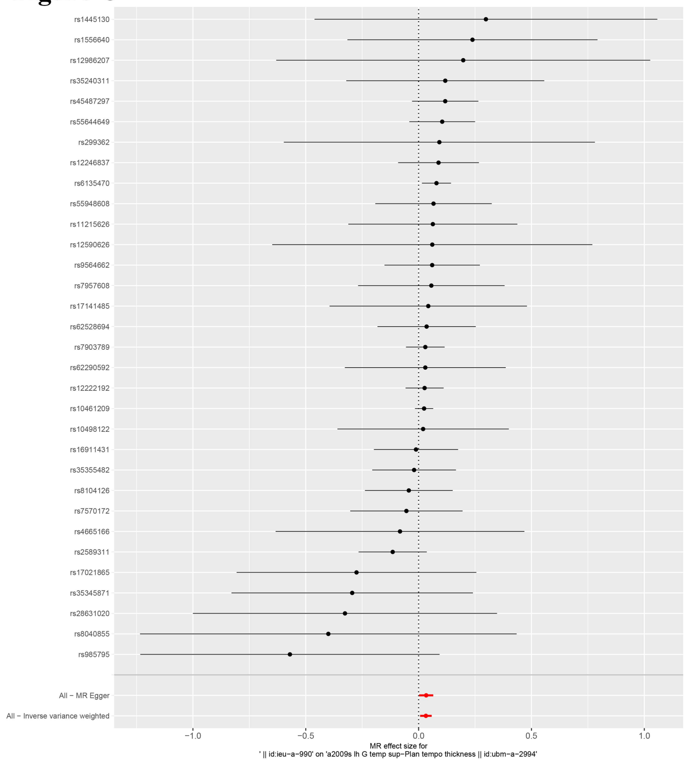

Figure D

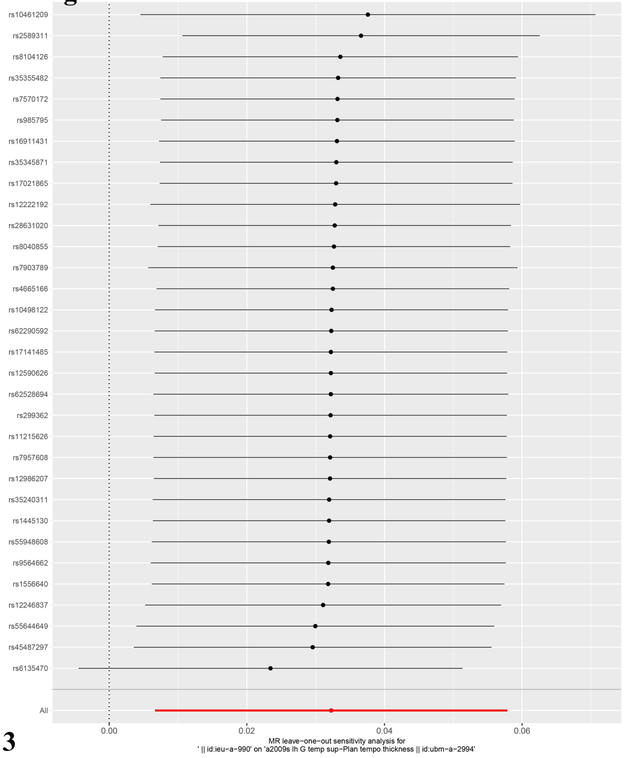

Figure A

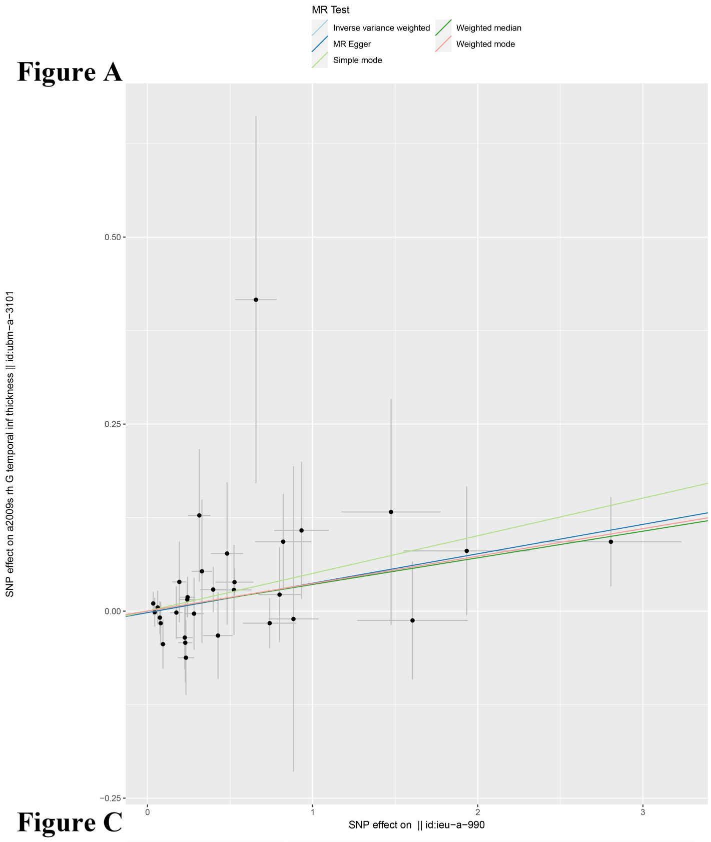

Figure B

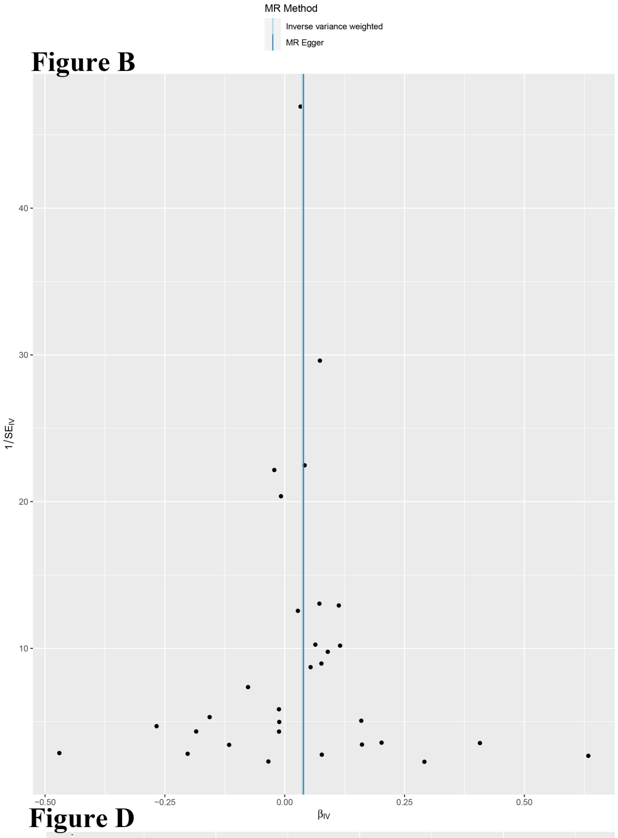

Figure C

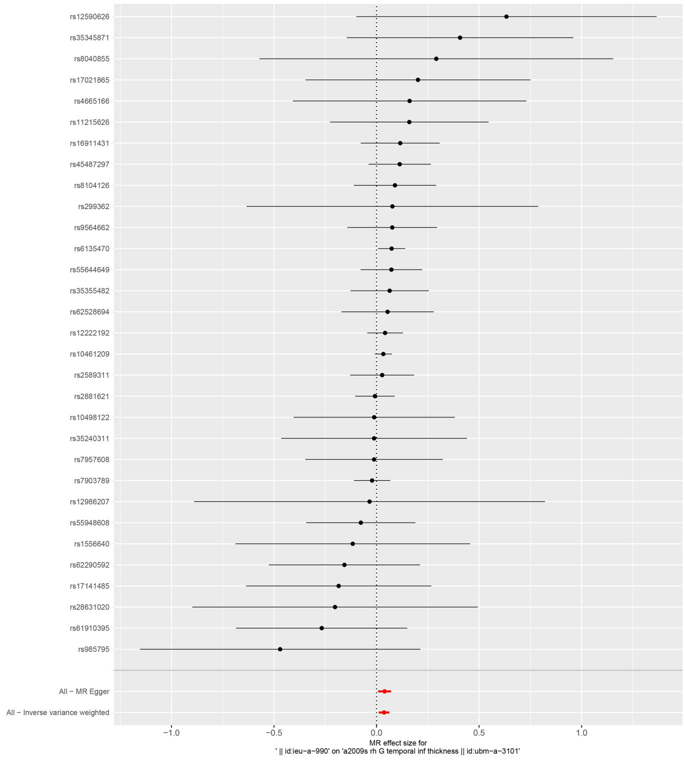

Figure D

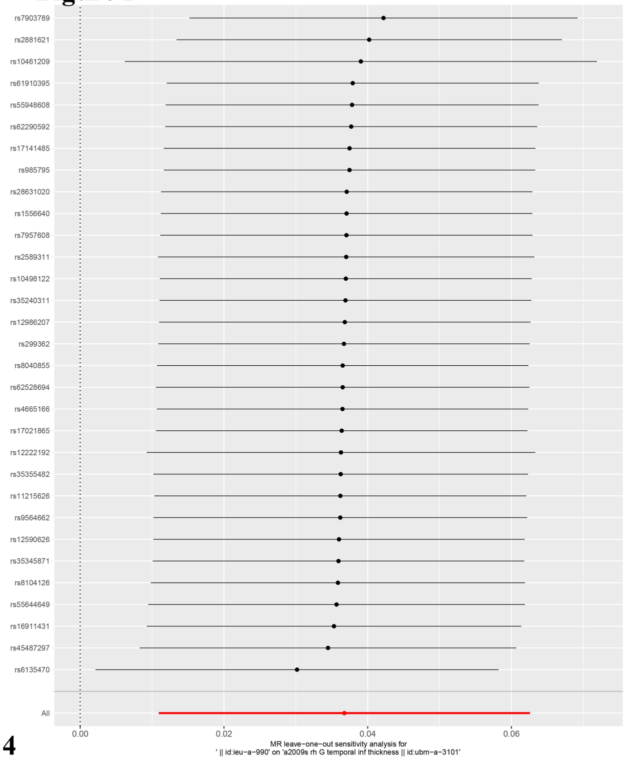

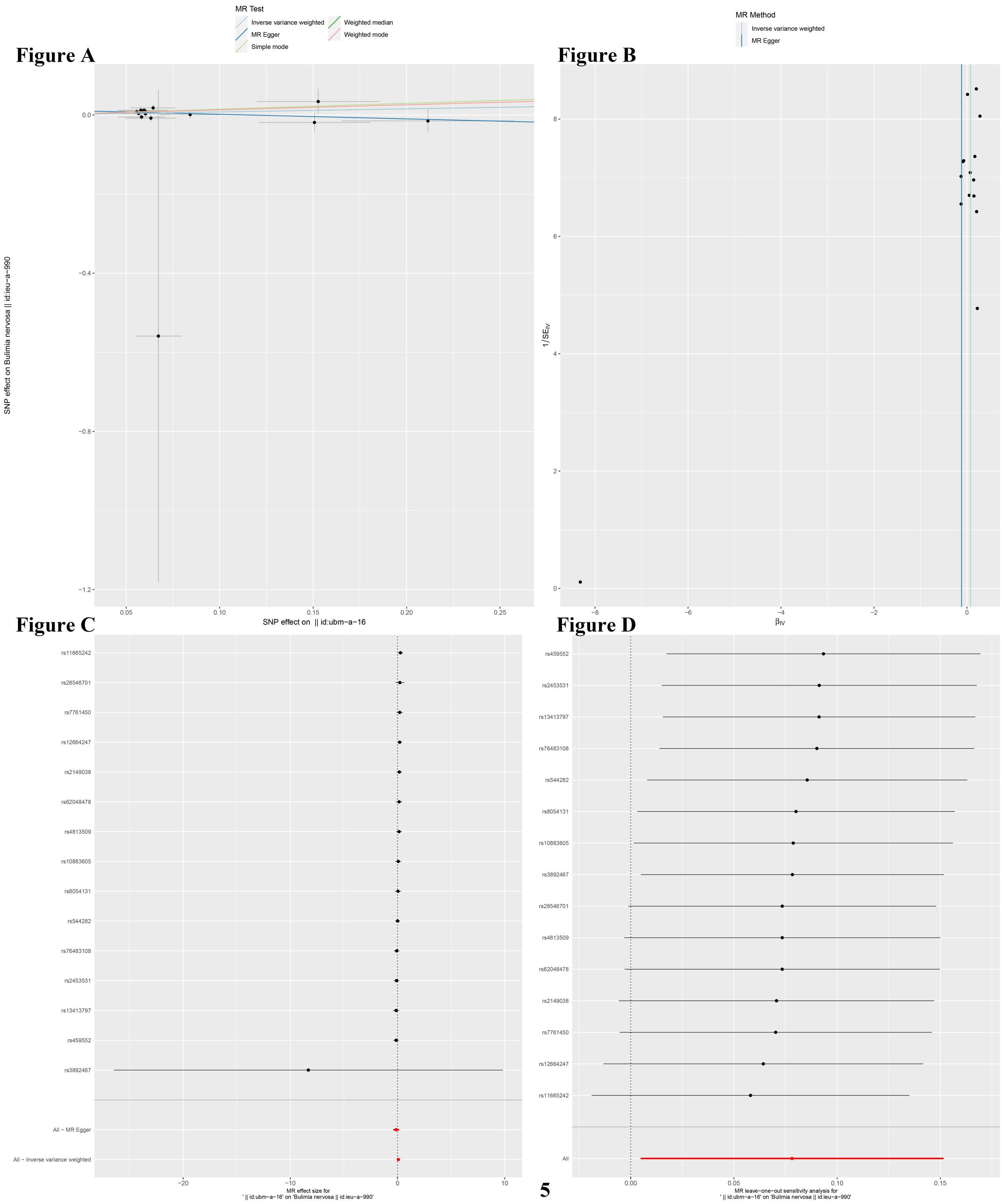
Figure A
MR Test
Inverse variance weighted
MR Egger
Simple mode
Weighted median
Weighted mode
SNP effect on Bulimia nervosa || id:ieu-a-990
SNP effect on || id:ubm-a-16
Figure B
MR Method
Inverse variance weighted
MR Egger
1/SE_IV
β_IV
Figure C
rs11665242
rs28546701
rs7761450
rs12664247
rs2149038
rs62048478
rs4813509
rs10883605
rs8054131
rs544282
rs76483108
rs2453531
rs13413797
rs459552
rs3892467
All − MR Egger
All − Inverse variance weighted
MR effect size for
' || id:ubm-a-16' on 'Bulimia nervosa || id:ieu-a-990'
Figure D
rs459552
rs2453531
rs13413797
rs76483108
rs544282
rs8054131
rs10883605
rs3892467
rs28546701
rs4813509
rs62048478
rs2149038
rs7761450
rs12664247
rs11665242
All
MR leave−one−out sensitivity analysis for
' || id:ubm-a-16' on 'Bulimia nervosa || id:ieu-a-990'

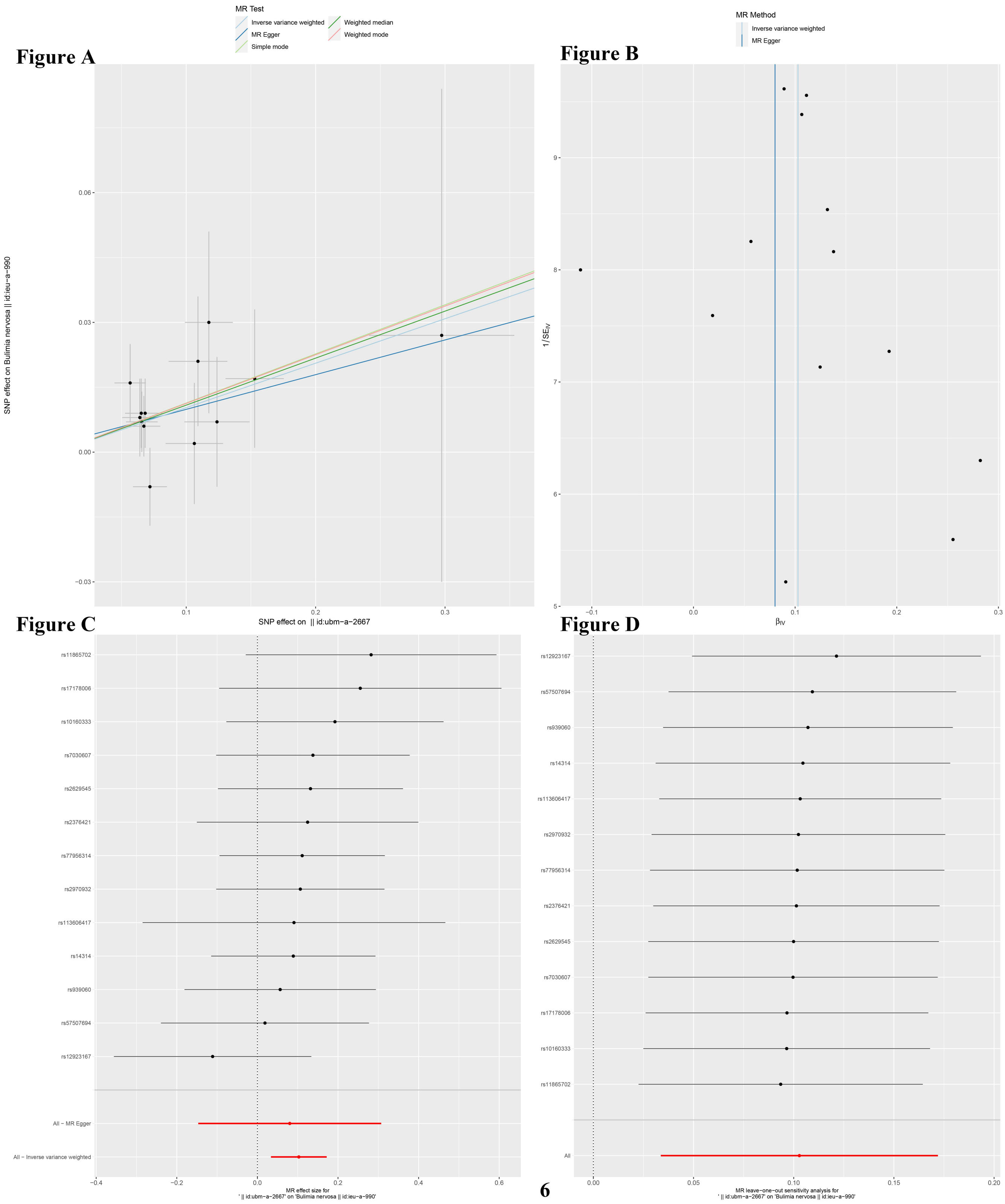
Figure A
MR Test
Inverse variance weighted
MR Egger
Simple mode
Weighted median
Weighted mode
SNP effect on Bulimia nervosa || id:ieu-a-990
SNP effect on || id:ubm-a-2667
Figure B
MR Method
Inverse variance weighted
MR Egger
1/SE_IV
β_IV
Figure C
rs11865702
rs17178006
rs10160333
rs7030607
rs2629545
rs2376421
rs77956314
rs2970932
rs113606417
rs14314
rs939060
rs57507694
rs12923167
All - MR Egger
All - Inverse variance weighted
MR effect size for
' || id:ubm-a-2667' on 'Bulimia nervosa || id:ieu-a-990'
Figure D
rs12923167
rs57507694
rs939060
rs14314
rs113606417
rs2970932
rs77956314
rs2376421
rs2629545
rs7030607
rs17178006
rs10160333
rs11865702
All
MR leave-one-out sensitivity analysis for
' || id:ubm-a-2667' on 'Bulimia nervosa || id:ieu-a-990'

6

Figure A

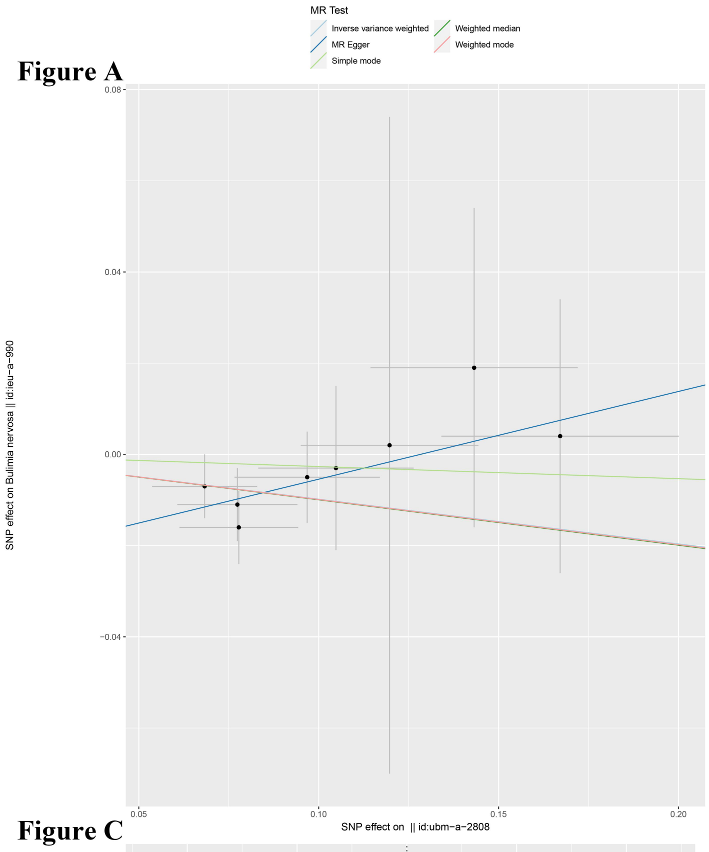

Figure B

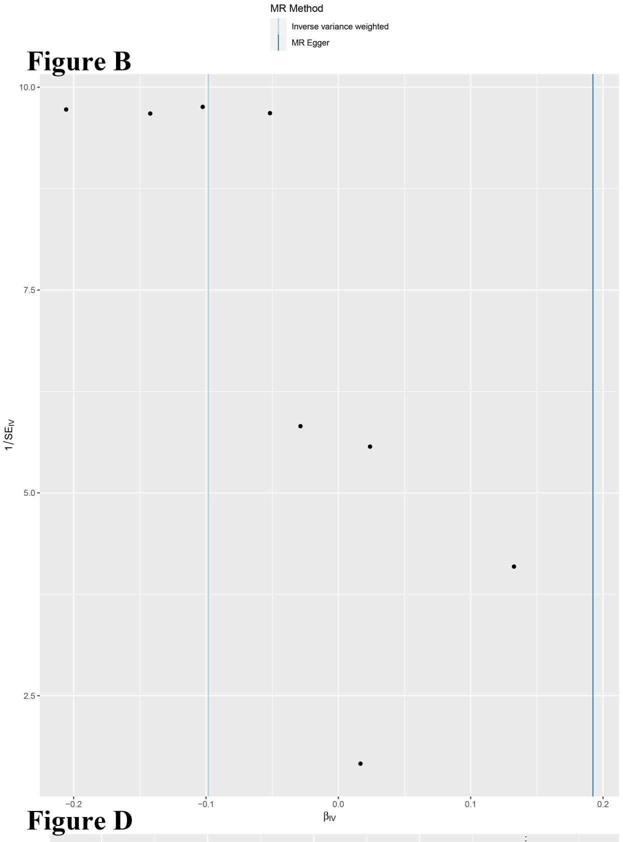

Figure C

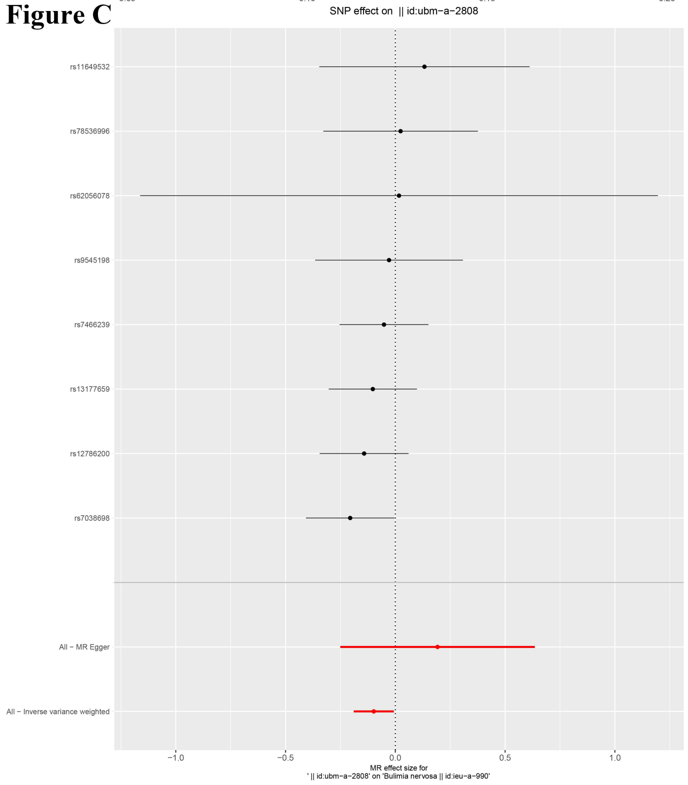

Figure D

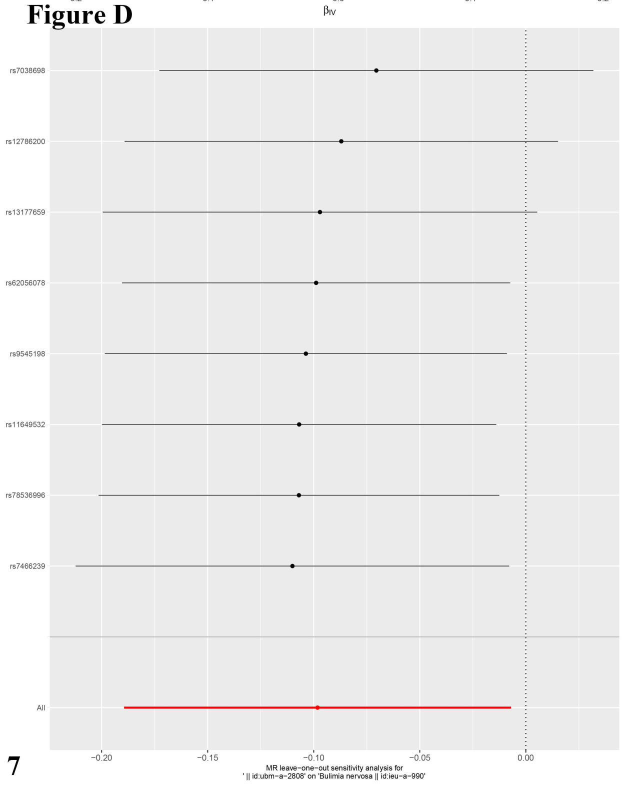

Figure A

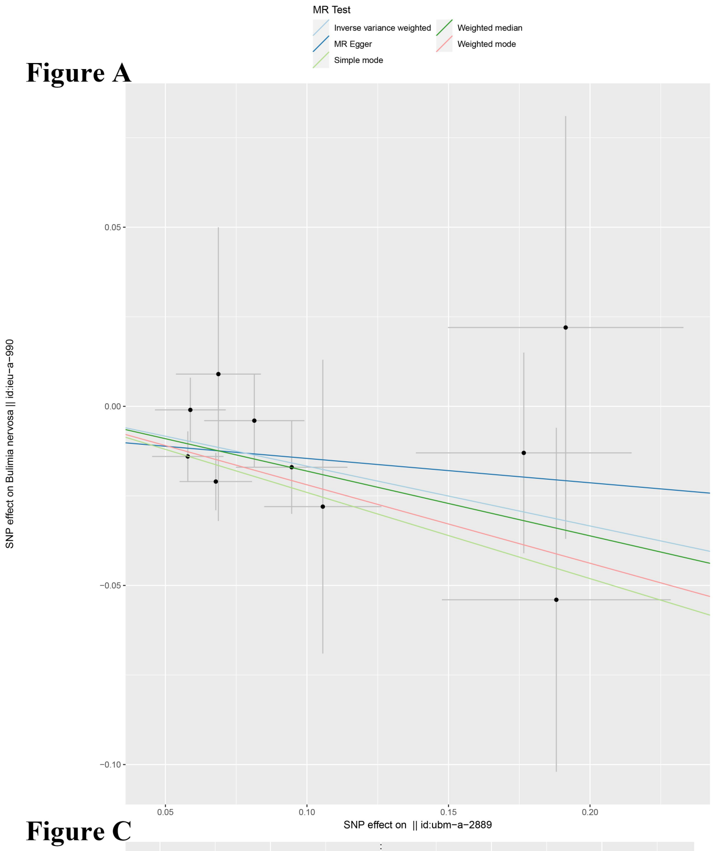

Figure B

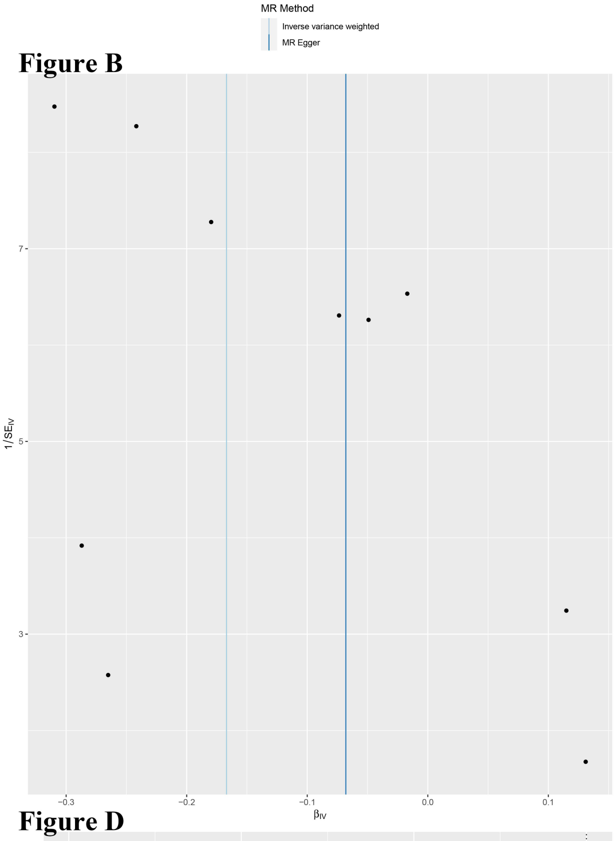

Figure C

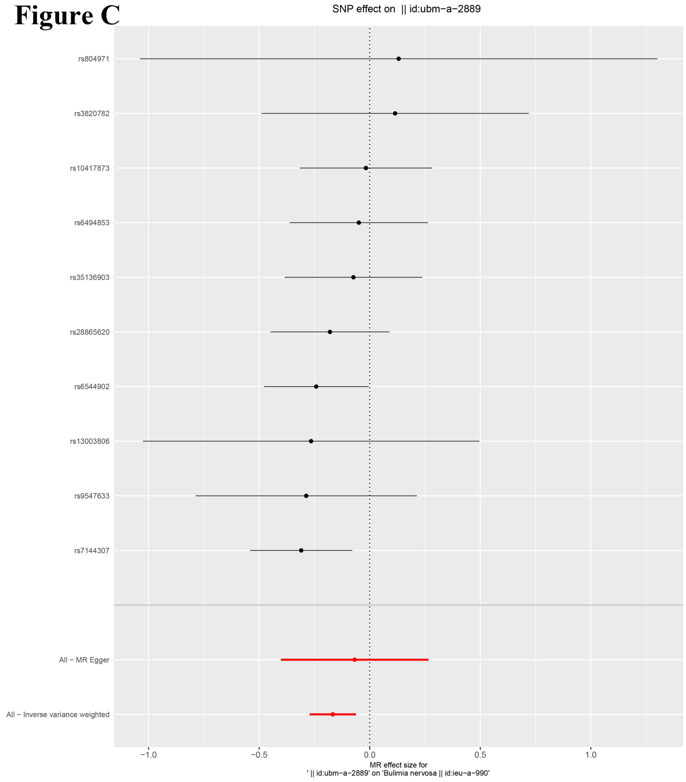

Figure D

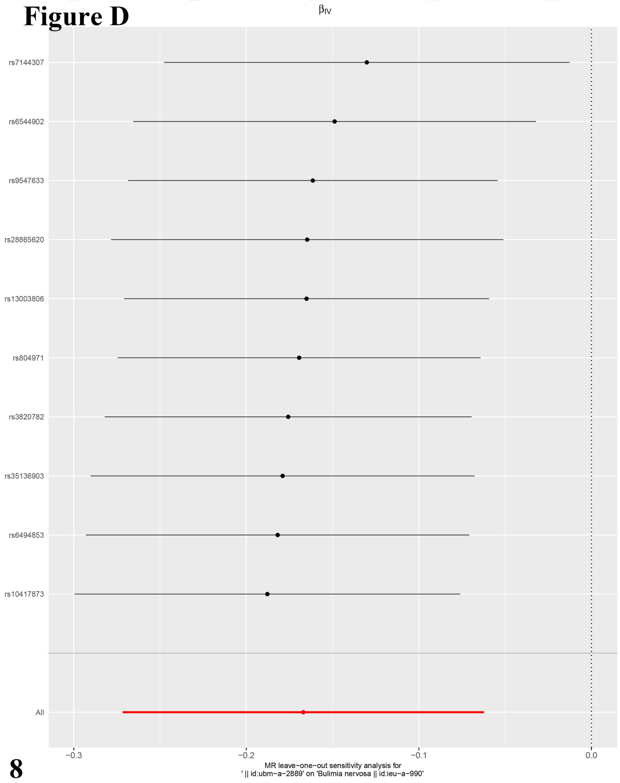

Figure A

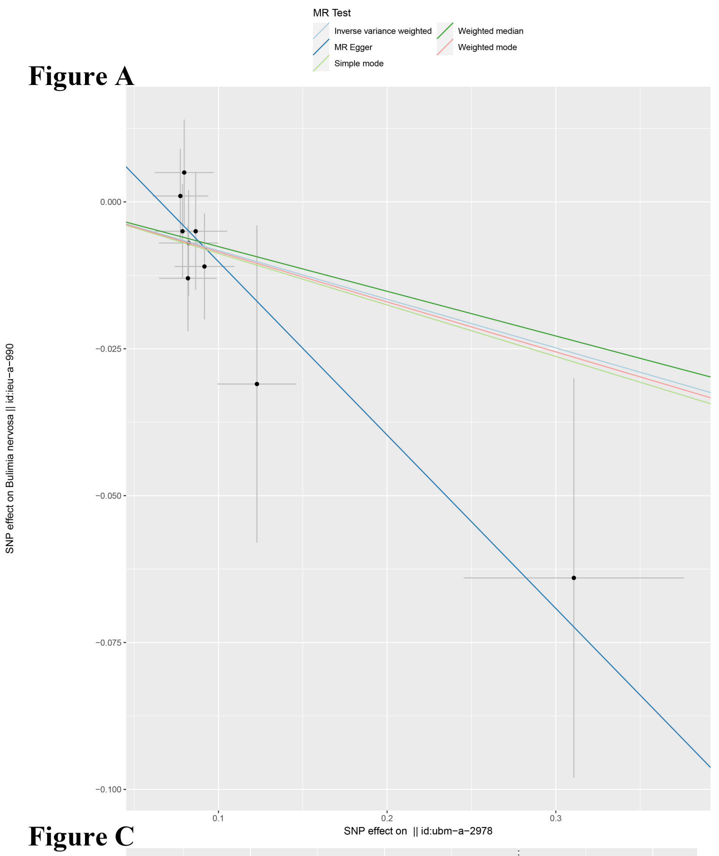

Figure B

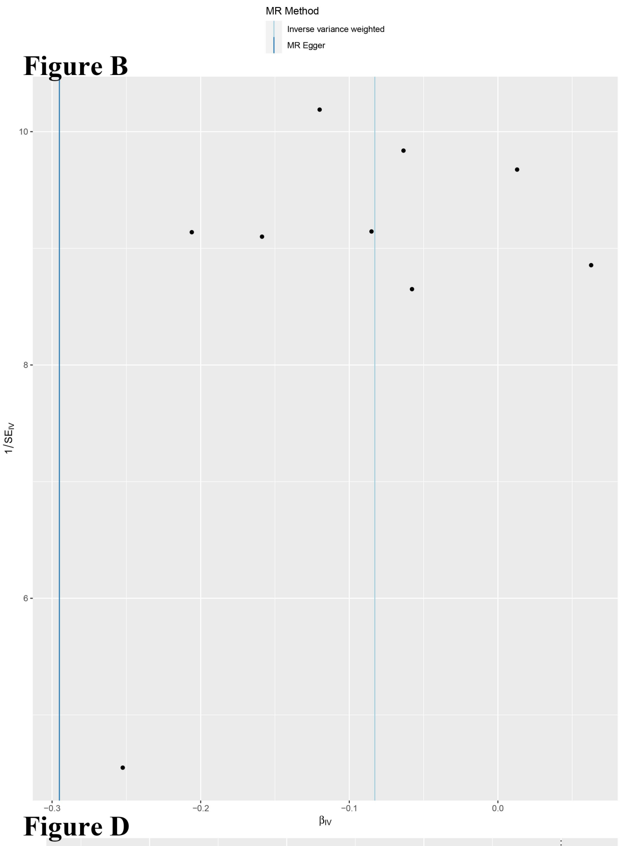

Figure C

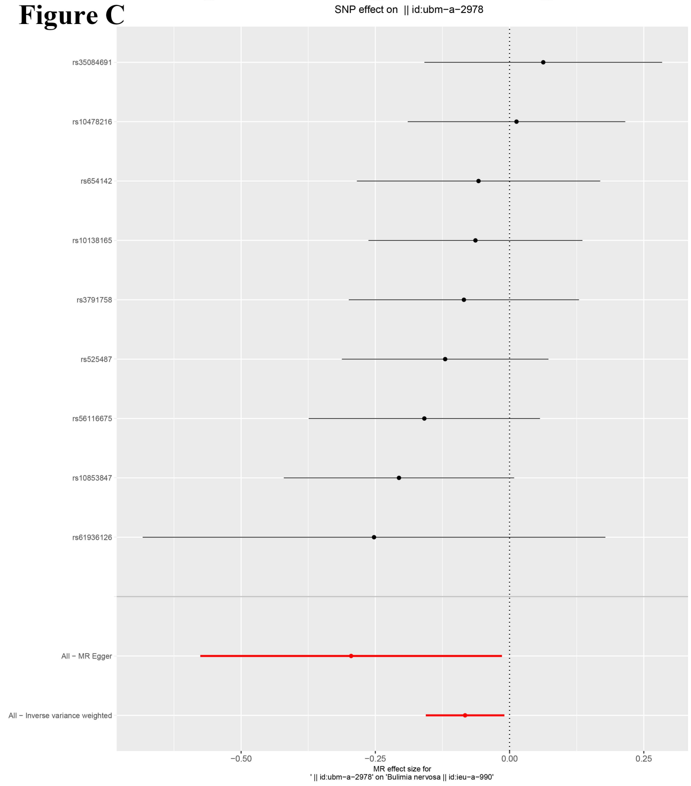

Figure D

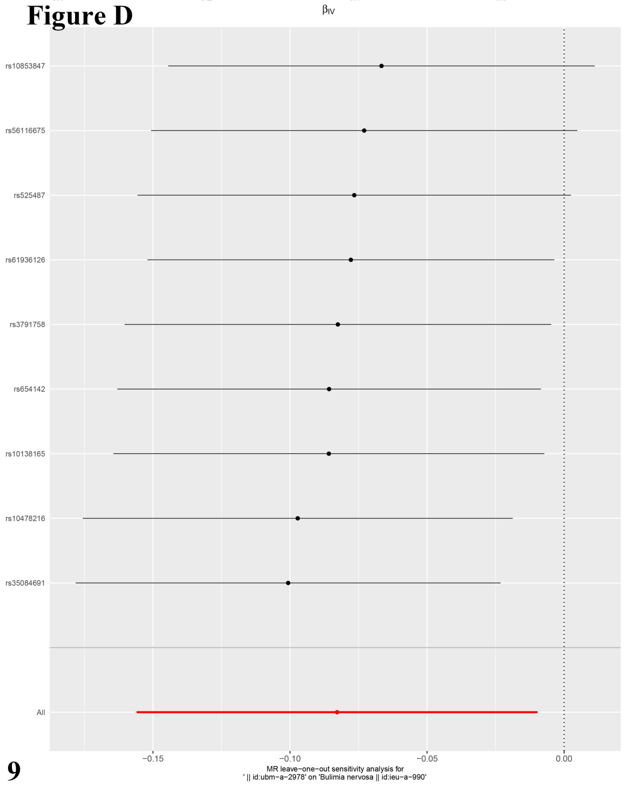

Figure A

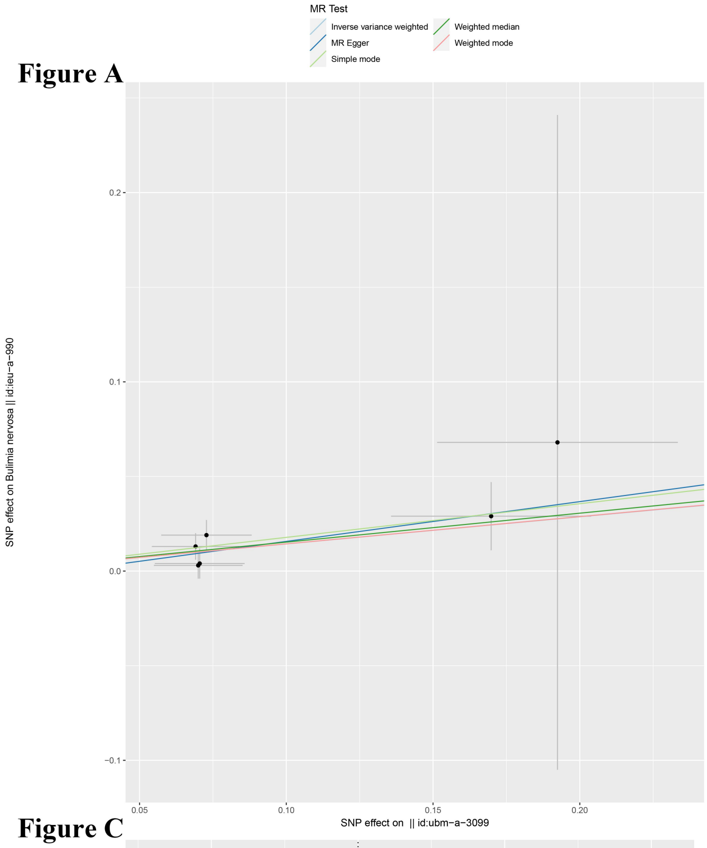

Figure B

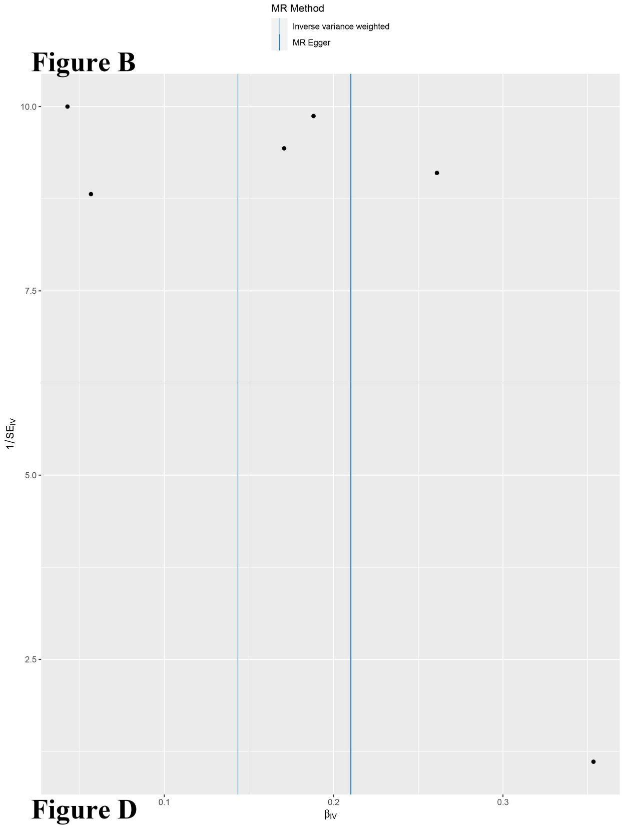

Figure C

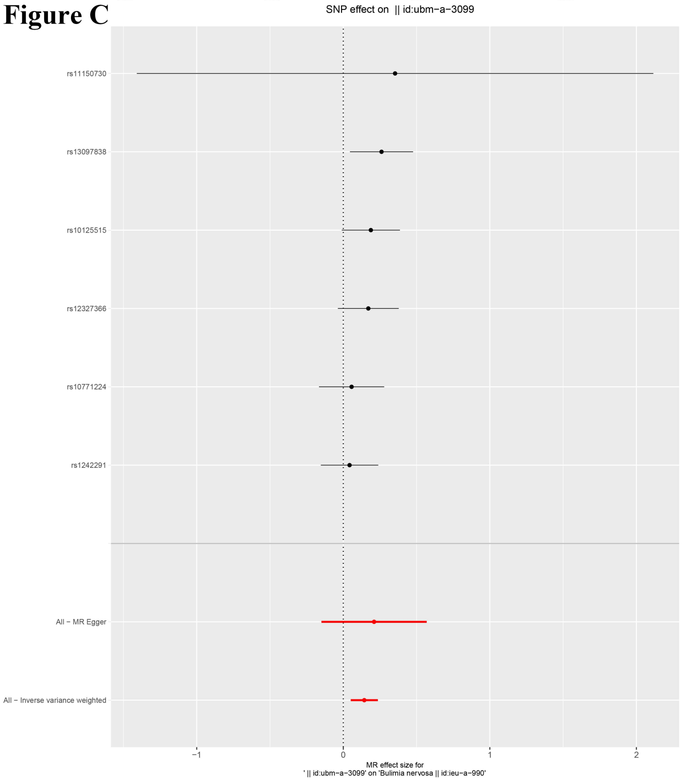

Figure D

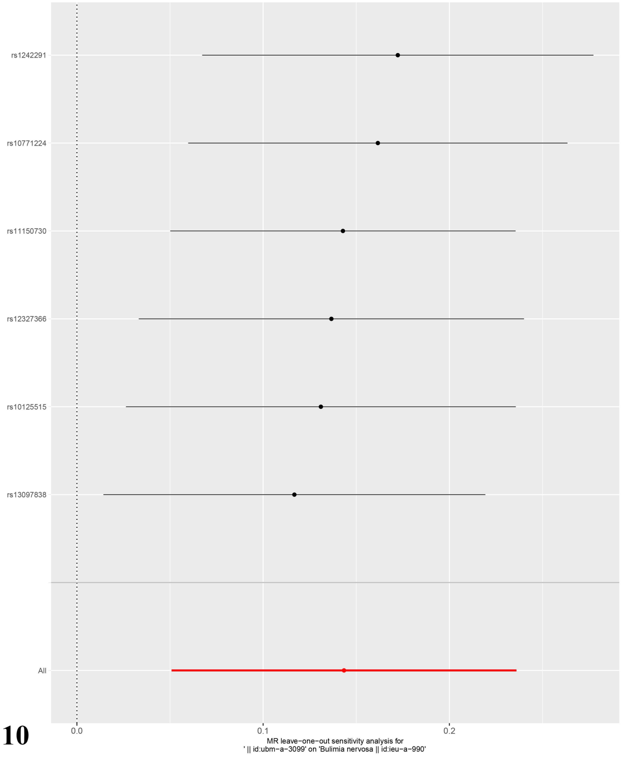

Supplement: Supplementary file 6 — Supplementary Material: brb370859‐sup‐0006‐SuppMat6.pdf [file BRB3-15-e70859-s004.pdf]
